# A Novel Tetrahydroacridine Derivative with Potent Acetylcholinesterase Inhibitory Properties and Dissociative Capability against Aβ42 Fibrils Confirmed by In Vitro Studies

**DOI:** 10.3390/ijms251810072

**Published:** 2024-09-19

**Authors:** Ilona Mojzych, Anna Zawadzka, Kryspin Andrzejewski, Monika Jampolska, Zuzana Bednarikova, Miroslav Gancar, Zuzana Gazova, Maciej Mazur, Katarzyna Kaczyńska

**Affiliations:** 1 Department of Chemistry, University of Warsaw, Pasteura 1, 02-093 Warsaw, Poland; ilona.mojzych@student.uw.edu.pl (I.M.); azawadzka@chem.uw.edu.pl (A.Z.); mmazur@chem.uw.edu.pl (M.M.); 2 Department of Respiration Physiology, Mossakowski Medical Research Institute, Polish Academy of Sciences, Pawińskiego 5, 02-106 Warsaw, Poland; kandrzejewski@imdik.pan.pl (K.A.); mjampolska@imdik.pan.pl (M.J.); 3 Department of Biophysics, Institute of Experimental Physics, Slovak Academy of Sciences, 04001 Kosice, Slovakia; bednarikova@saske.sk (Z.B.); gancar@saske.sk (M.G.); gazova@saske.sk (Z.G.)

**Keywords:** Alzheimer’s disease, acetylcholinesterase inhibitor, therapy, Aβ fibrils

## Abstract

Alzheimer’s disease (AD) is one of the most common causes of dementia, accounting for more than 60% of all cases. It is a neurodegenerative disease in which symptoms such as a decline in memory, thinking, learning, and organizing skills develop gradually over many years and eventually become more severe. To date, there is no effective treatment for the cause of Alzheimer’s disease, and the existing pharmacological options primarily help manage symptoms. Treatment is mainly based on acetylcholinesterase (AChE) inhibitors such as donepezil, rivastigmine, and galantamine, which exhibit numerous adverse cardiovascular and gastrointestinal effects due to excessive stimulation of peripheral cholinergic activity involving muscarinic receptors. Therefore, in addition to the obvious drugs that act on the cause of the disease, new drugs based on AChE inhibition that show the fewest side effects are needed. One potential drug could be a new compound under study, tetrahydroacridine derivative (CHDA), which showed significant potential to inhibit the AChE enzyme in previous in vitro studies. The present study shows that while having very potent AChE inhibitory properties, CHDA is a compound with low toxicity to nerve cell culture and living organisms. In addition, it exhibits dissociative activity against amyloid β fibrils, which is extremely important for applications in Alzheimer’s disease therapy.

## 1. Introduction

Alzheimer’s disease (AD) is a form of brain degeneration that progressively compromises memory and cognitive abilities because of the selective loss of vulnerable brain areas. The underlying cause of brain neuronal death is the deposition and aggregation of amyloid β (Aβ) protein into extracellular senile plaques and intracellular microtubule-associated tau protein into neurofibrillary tangles (NFTs). Abnormal protein folding, cerebral vascular damage, neuroinflammation, blood–brain barrier (BBB) damage, oxidative stress, mitochondrial dysfunction that leads to synaptic dysfunction, and neuronal loss are all associated with AD initiation and progression [1,2]. It has been assessed that one in nine people aged 65 and older is affected by Alzheimer’s disease and that this number will gradually increase [3]. Despite the advancing knowledge of the pathomechanisms of the disease, its treatment is mainly symptomatic. Pharmacological intervention comprises the NMDA receptor antagonist, memantine, and three acetylcholinesterase (AChE) inhibitors such as donepezil, rivastigmine, and galantamine. Memantine, by inhibiting the activity of the NMDA receptor, diminishes the effects of pathologically increased glutamate concentrations, which cause neurotoxicity and neuronal degradation [4,5]. The mainstay of treatment, however, is medication that leads to an increase in cholinergic transmission. In Alzheimer’s disease, there is selective destruction of cholinergic neurons located in the basal forebrain, and damage to the cholinergic system, responsible for arousal and attention, correlates with the severity of memory loss. Therefore, AChE inhibitors that stop the enzyme that breaks down one of the main neurotransmitters, acetylcholine, are used. This causes an increase in acetylcholine levels in the cholinergic synapses of the central nervous system, which to some extent compensates for the loss of cholinergic neurons and has a beneficial effect on the treatment of mild to moderate dementia [6,7]. AChE inhibitors, while slowing the progression of cognitive impairment moderately [8,9], are also characterized by numerous side effects, such as nausea, vomiting, salivation, diarrhea, weight loss, and muscle weakness [7]. AD therapy has recently been enriched with the first disease-modifying drugs, based on monoclonal antibodies against amyloid, approved by the Food and Drug Administration [7,10]. Used in the early stages of the disease, they provide moderate benefits, but they have not yet been approved worldwide as they require additional clinical data to confirm their efficacy and safety. As a result, attempts are still being made to develop new formulations based on inhibition of the AChE enzyme to reduce their undesirable effects or improve their bioavailability. In the present work, we addressed a new tetrahydroacridine derivative (CHDA), the electrochemical properties of which were studied in our recent work, which exhibits acetylcholinesterase inhibitory activity [11]. The latter characteristic opens the prospect of a potential application of the CHDA compound in the treatment of Alzheimer’s disease. The aim of the present study was to investigate the properties of this compound that would indicate its lack of toxicity and potential usefulness in AD therapy. Accordingly, the following properties were determined: in vitro and in vivo AChE inhibition after systemic application of CHDA, its effect on Aβ42 fibril degradation, neuronal cell viability, and organ toxicity under repeated exposure.

## 2. Results and Discussion

### 2.1. Inhibition of Acetylcholinesterase (AChE) Activity—In Vitro Studies

**Figure 1:**
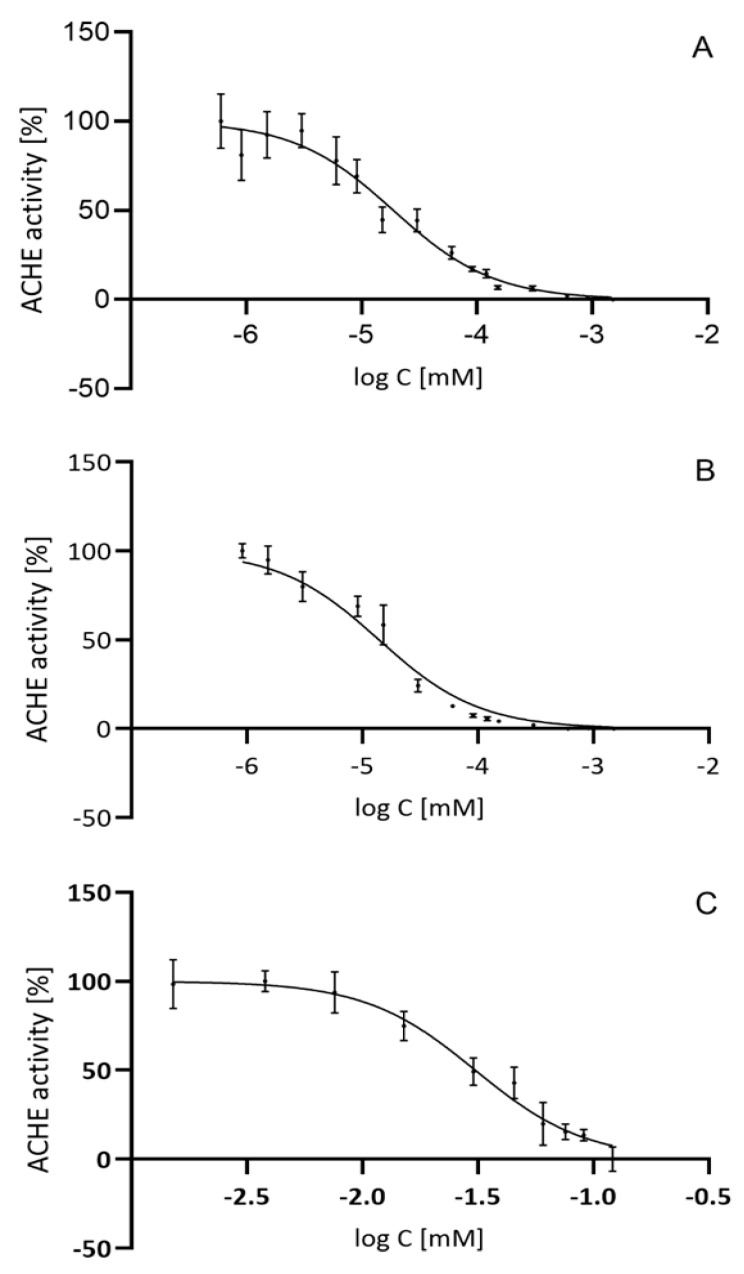
CHDA and liposomal CHDA effectively inhibit AChE activity. Different concentrations of CHDA (**A**), liposomal CHDA (**B**), and rivastigmine (**C**) were added to the test solution and incubated with AChE, after which ATCI was added to the solution. AChE activity and IC50 values were calculated. Each point is the average of eight replicates of the assay (mean ± SD).

Treatment for Alzheimer’s disease, which is still limited and mainly symptomatic, uses drugs that increase the activity of the cholinergic system, specifically in the early stages of the disease. Therefore, inhibitors of AChE, an enzyme that breaks down one of the primary neurotransmitters, acetylcholine, are used. This increases the level of acetylcholine in the cholinergic synapses of the central nervous system, with beneficial effects in the treatment of dementia [12,13]. Inhibitors used in clinical practice have numerous side effects [14,15], hence the need for new compounds that act similarly but with fewer unwanted effects. An in vitro study was therefore conducted to confirm the inhibitory effect of CHDA on the AChE enzyme from *Electrophorus electricus*. The enzyme’s inhibitory potency was compared with liposomal CHDA and with a reference compound, rivastigmine. The dose–effect curves used to estimate the IC50 index, which determines the concentration of the inhibitor that causes 50% inhibition of the AChE enzyme, are shown in Figure 1.

The calculated IC50 for CHDA of 18.55 ± 5.68 nM is three orders of magnitude lower than that for rivastigmine (30.85 ± 8.8 µM); this is a very promising result that validates our previous findings [11]. On the other hand, a comparison of the IC50 for liposomal CHDA (IC50 = 13.92 ± 1.75 nM) with the CHDA result shows that the use of liposomes does not worsen the inhibitory properties of the AChE enzyme, and, in fact, it reduces this value by 25%. Overall, CHDA inhibits the AChE enzyme at much lower concentrations than rivastigmine, which, with the potential use of the compound in the clinic, would allow for a significant reduction in the dose of the inhibitor. In addition, CHDA shows more potent inhibitory activity compared with recently described, newly designed tacrine-derived cholinesterase inhibitors, with IC50 values ranging from 117.5 to 455 nM for AChE [16].

### 2.2. Effects on the Degradation of Aβ42 Protein Fibrils

**Figure 2:**
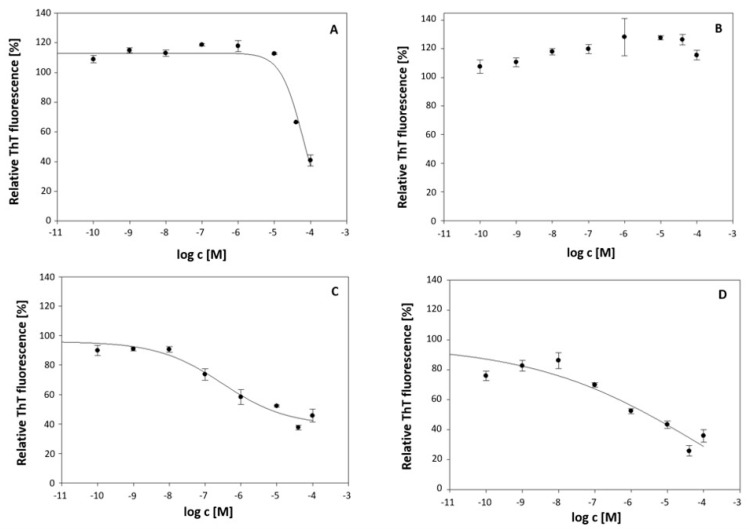
Effect of the dissociation of CHDA (**A**), rivastigmine (**B**), liposomes (**C**), and liposomal CHDA (**D**) on Aβ42 fibrils (5 μM) in the concentration range of 100 pM to 100 µM determined by the thioflavin T fluorescence assay. The measured ThT fluorescence intensities of the samples were normalized to the ThT fluorescence intensity detected for untreated Aβ42 fibrils taken as 100%. Data represent the mean of three independent samples ± standard deviation.

In the area of drug development for Alzheimer’s disease, data are lacking on their potential to dissociate amyloid protein aggregates. To determine their potential against full-length Aβ42 peptide, fibrils were incubated with various concentrations of AChE inhibitors. Aβ42 peptide fibrils are a major component of senile plaques, a neuropathological diagnostic feature fundamental to AD, and a major target of current antibody-based therapies acting on the cause of AD. Mature Aβ42 fibrils were treated with the formulations at a wide range of concentrations from 100 pM to 100 µM for 24 h. In vitro studies using the thioflavin T fluorescence assay demonstrated the ability of all the tested compounds except rivastigmine to dissociate and thus reduce the amount of Aβ42 fibrils (Figure 2). The dissociation effect of the tested formulations was dose-dependent, but the degree of this effect for each formulation varied. Rivastigmine (Figure 2B) showed no ability to destroy Aβ42 fibrils in the range of concentrations used, while the other compounds tested caused a decrease in ThT fluorescence intensity compared with that of untreated fibrils. DC50 values corresponding to concentrations with 50% dissociation activity were determined for CHDA, liposomal CHDA, and liposomes alone. The lowest DC50 values were obtained for liposomes (3 μM) and liposomal CHDA (6 μM), indicating the highest dissociation potential. There is evidence that Aβ42 fibrils can interact with and may even be disrupted by liposomes, utilizing so-called “active” water, which aligns well with our observed results [17,18,19]. CHDA alone had milder activity with a DC50 of 70.8 μM. This shows that loading CHDA into liposomes significantly increases the dissociative potential of the compound. Vu et al. reported a similar phenomenon, where the presence of liposomes was observed to enhance the effect of dopamine against Aβ42 fibrils [19].

**Figure 3:**
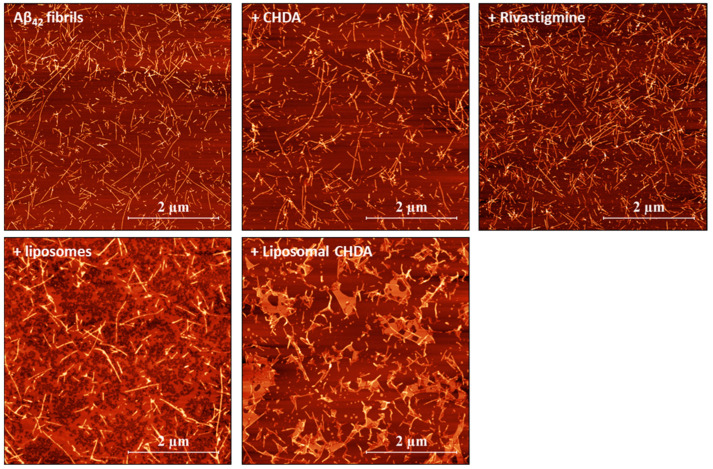
Representative images of Aβ42 fibrils (5 μM concentration) without and after a 24 h treatment with CHDA, rivastigmine, liposomal CHDA at a 40 μM concentration, and liposomes alone. The images are 5 × 5 µm at a 1024 × 1024-pixel resolution.

The activity of the tested formulations and the morphology of Aβ42 amyloid aggregates were imaged with atomic force microscopy (Figure 3). Aβ42 peptide formed a dense network of thin fibrils with a diameter of ~10 nm and a length of several μm under appropriate conditions. A twenty-four-hour treatment with the test formulations at a concentration of 40 μM led to a reduction in the number of Aβ42 fibrils and changes in their morphology. Such a reduction in the number of fibrils was observed after treatment with liposomes and liposomal CHDA. In the case of liposomal CHDA, lipids from the liposomes were most likely visible, obscuring the fibrils. In contrast, in the presence of ineffective rivastigmine, the number of Aβ42 fibrils appeared to be increased. 

At the chosen concentration of 40 μM, CHDA had milder dissociation activity than liposomal CHDA, which was also visualized by AFM with more fibrils still present in the sample. The AFM supports the ThT assay results, suggesting that liposomal CHDA exhibits significant dissociative potential against Aβ42 fibrils, even surpassing the CHDA alone. Compared with rivastigmine, CHDA not only showed AChE enzyme inhibitor properties but also showed dissociative properties of amyloid Aβ fibrils, a feature highly desirable in the search for new therapies for Alzheimer’s disease. CHDA appears to have the potential to be a compound that acts on both the cause of the disease and its effect.

### 2.3. In Vitro Toxicity—Effects on Nerve Cells

**Figure 4:**
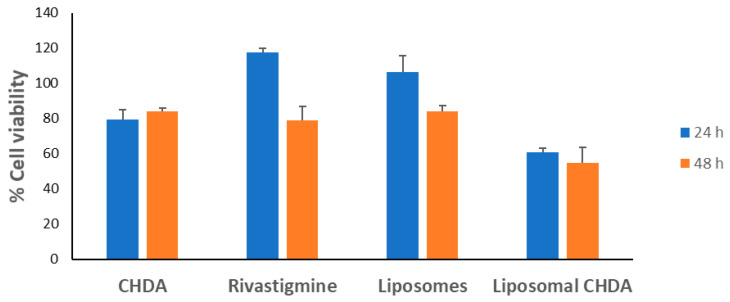
SH-SY5Y cell viability (%) after 24 and 48 h of incubation with CHDA, rivastigmine, liposomal CHDA (10 μM), and liposomes alone determined using the WST-1 assay. All data are representative of 2–3 independent experiments ± SD. Data obtained were normalized to absorbance taken as 100%, measured for control cells not treated with test compounds. Statistical analysis showed no significant differences among the study groups.

A compound used in the treatment of neurodegenerative diseases is not supposed to be toxic to nerve cells, so the cytotoxicity of CHDA on the SH-SY5Y neuroblastoma cell line was tested and compared with the cytotoxicity of rivastigmine, liposomal CHDA, and liposomes alone. Cells were treated with 10 μM concentrations of the test compounds for 24 and 48 h, and the effect on proliferation was determined using the WST-1 assay (Figure 4). After 24 h of incubation, the proliferation of SH-SY5Y cells was inhibited by 21% by CHDA and by 39% by liposomal CHDA compared with untreated cells taken as 100%. The rivastigmine and liposomes slightly increased the cell viability. The 48 h treatment did not affect cell viability, which was about 80% for CHDA, rivastigmine, and liposomes. The lowest viability of neuroblastoma cells of about 60% at both 24 and 48 h was observed for liposomal CHDA, which is somewhat surprising, as both components used separately inhibit the survival of SH-SY5Y cells to a lesser extent. Moreover, liposome encapsulation is used to protect against the toxic effects of various bioactive agents, and liposomes themselves are considered to be low in toxicity [20,21]. Nevertheless, the survival rate is at an acceptable level, and statistical analysis showed no significant differences between the study groups.

### 2.4. In Vivo Subacute Toxicity

The possible medical applications of the CHDA compound or its liposomal form require that they must be non-toxic when administered into the body. To this end, the next step was to study the toxicity of CHDA and its liposomal form administered by intraperitoneal injection for seven consecutive days. The subacute toxicity of the compounds was compared with a group treated identically with a physiological salt solution (NaCl) and rivastigmine, a reference compound used to treat Alzheimer’s disease. All blood and urine analysis data indicated no toxicity of the CHDA compound or its liposomal form. Seven days after the end of the injection, baseline hematological parameters such as leukocytes (WBCs), erythrocytes (RBCs), hemoglobin (HGB), hematocrit (Hct), and platelets (PLTS), as well as the others listed in Table 1, were within the normal range in all study groups. The only significantly different parameters were hemoglobin and erythrocyte volume distribution coefficients of variation (RDW-CV), which were significantly lower in rats with rivastigmine or CHDA in liposome form compared with the CHDA group (Table 1). RDW-CV is a parameter that has little diagnostic significance if the other morphology parameters are within accepted norms [22,23]. Importantly, hemoglobin in all groups was within the range of accepted norms [24,25], and both RDW-CV and hemoglobin were not significantly different in any of the groups tested with AChE inhibitors compared to the control group. The high platelet content in rats (500–1300 G/L) compared with humans (140–600 G/L) is a physiological phenomenon [26]. Levels of urea (BUN) and creatinine (CREA), biomarkers of kidney damage [27] that can indicate changes in glomerular filtration rate or problems with urine elimination [28], were not elevated. Similarly, the levels of both alanine aminotransferase (ALT) and aspartate transaminase (AST), enzymes that are major indicators of liver function and health [29], were not significantly increased compared to the control group, demonstrating the absence of hepatocyte damage [30]. CHDA appears to be safe in contrast to, for example, tacrine, which is a cholinesterase inhibitor and the first drug approved for the treatment of AD, but it was withdrawn because of its hepatotoxicity [31]. Clearly, further studies are needed to test chronic exposure to the CHDA compound to rule out its hepatotoxicity and other side effects. Additional tests such as arterial blood gas analysis also showed no significant changes among the study groups (Table 1). Urine analysis (Table 2) showed no urinary tract infection or renal dysfunction in any study group. In addition, the absence of glucose in the urine indicates undisturbed pancreatic function [32]. Increased urinary protein values in all groups of animals, including after administration of AChE enzyme inhibitors, are due to the physiological phenomenon of increased glomerular capsule size in male Wistar rats [33].

**Figure 5:**
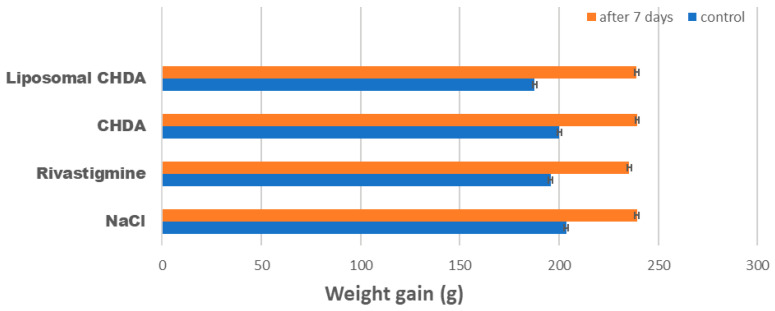
Weight gain of animals after 7 days of intraperitoneal injections of NaCl, rivastigmine, CHDA, and liposomal CHDA (n = 6 per group). Statistical analysis showed no significant differences in weight gain among the study groups.

In addition, the body weight of rats administered CHDA or liposomal CHDA increased after seven days of injection similarly to the control rats by an average of thirty-odd grams, indicating no deterioration in animal welfare or evidence of toxicity (Figure 5). These preliminary repeated exposure subacute toxicity results for the CHDA compound and its liposomal form showed no adverse effects on any of the parameters tested and can therefore be considered potentially safe. However, further studies are needed to provide chronic toxicity data for CHDA.

### 2.5. Acetylcholinesterase (AChE) Activity in Hippocampal Homogenates after Injection of CHDA, Liposomal CHDA, and Rivastigmine

**Figure 6:**
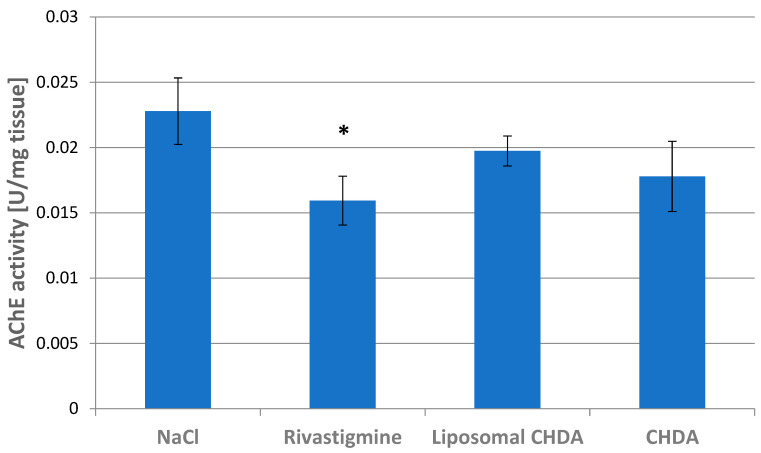
Acetylcholinesterase activity in the hippocampus of rats that received intraperitoneal injection of NaCl, rivastigmine, CHDA, and liposomal CHDA. Data are expressed in mU min^−1^ mg^−1^ and presented as mean ± SD, * *p* < 0.05, (n = 4 in each group).

An important goal of the present study was to see if the formulation under investigation is able to inhibit AChE activity in brain tissue after systemic administration, which would demonstrate its ability to penetrate the blood–brain barrier (BBB). This property, which is extremely important in neurodegenerative diseases of the brain, was compared with CHDA administered in liposomal form, designed to facilitate the compound’s reaching the brain, and with rivastigmine, an AChE inhibitor used to treat dementia associated with Alzheimer’s disease. Acetylcholinesterase activity in the hippocampus was evaluated in four groups of rats given NaCl, rivastigmine, CHDA, and CHDA in liposomal form by intraperitoneal injection. The rats that received rivastigmine had 31% significantly lower AChE activity in the hippocampus than the control rats that received saline 50 min after injection (Figure 6). This is consistent with previous studies; although the experimental conditions differ in dose, time of measurement of enzyme activity, etc., the results show that intraperitoneally administered rivastigmine (0.6 mg/kg) inhibited AChE in the rat cerebral cortex by about 40% [34], and in healthy young volunteers, a single oral dose of rivastigmine (3 mg) caused maximal inhibition of AChE in the cerebrospinal fluid by 38% after 2.4 h [35].

**Figure 7:**
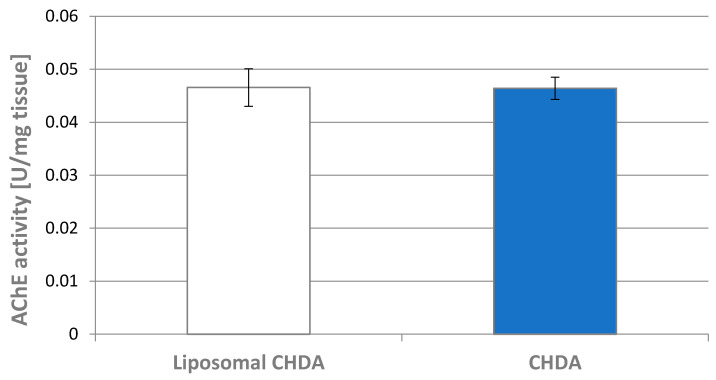
Acetylcholinesterase activity in the hippocampus of rats that received intravenous injections of CHDA and liposomal CHDA. Data are expressed in mU min^−1^ mg^−1^ and presented as mean ± SD, (n = 4 in each group). Statistical analysis showed no significant differences between the study groups.

After CHDA administration, a 22% difference in enzyme activity was observed compared with the control group. Given the three orders of magnitude higher AChE inhibitory capacity compared with rivastigmine obtained in vitro, one would expect a greater effect if the CHDA compound passed through the BBB. Yet, the use of liposomes as CHDA carriers in an in vivo application did not improve the compound’s effectiveness in inhibiting AChE activity, which was reduced by only 12% (Figure 6). Considering that the intraperitoneal administration of liposomes can have effects limited to the site of administration and that their appearance in circulation depends largely on their size and the material from which they are made [36], we decided to test intravenous administration, which directly delivers liposomes into the bloodstream. Along with the bloodstream, they can quickly reach the brain. We therefore compared AChE activity after intravenous administration of CHDA alone and its liposomal form (Figure 7). Unfortunately, there were no differences between the two groups.

The results indicate that CHDA, compared with rivastigmine, passes through the blood–brain barrier more weakly and it is not facilitated by liposomes. The blood–brain barrier is a polarized monolayer composed of microvascular endothelial cells, connected to each other by tight junctions, which includes the apical membrane facing the bloodstream and the basement membrane facing the brain tissue. The BBB has specific properties that allow it to transport substances selectively from the blood into the cerebrospinal fluid, limiting the penetration of harmful substances into the central nervous system (CNS) [37]. At the same time, this is a limitation for drug treatment in neurodegenerative diseases, including Alzheimer’s disease, Parkinson’s disease, multiple sclerosis, and epilepsy, among others. The lipophilic nature of lipid-based nanocarriers such as liposomes should facilitate BBB passage [38], while the lack of such an effect in the present study may be related to the lack of liposome functionalization, resulting in their binding to brain endothelial receptors and facilitating BBB passage. An example of such functionalization is the conjugation of lactoferrin (Lf) on the surface of the nanocarriers. Lf receptors are overexpressed on brain endothelial cells at the BBB, interfering with transcytosis, the transport of macromolecules from the apical to the basolateral endothelial cell membrane [38,39]. Recent studies have demonstrated the superiority of Lf-functionalized nanocarriers, improving the absorption of the drug across the BBB. The physicochemical properties of huperzine, which is an acetylcholinesterase inhibitor like CHDA, make it poorly available in the central nervous system. The intranasal administration of huperzine in nanoemulsion with Lf resulted in its increased accumulation in the CNS compared with the administration of the free drug or in emulsion without Lf [40,41]. Further research is needed to look for new opportunities for CHDA permeation across the BBB, if only through liposome functionalization.

## 3. Materials and Methods

### 3.1. Synthesis of CHDA (6-chloro-1,2,3,4,9,10,11,12-octahydro-[1,4]-diazepine-[5,6,7-kl]-acridine Monohydrochloride)

**Figure 8:**
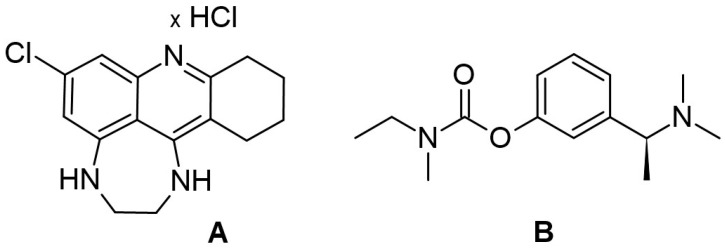
The chemical structures of CHDA (**A**) and rivastigmine (**B**).

The synthesis of the compound was described by us elsewhere [11]. The chemical structures of CHDA (A) and rivastigmine (B), as the reference compound, are shown in Figure 8.

### 3.2. Preparation of Neat and CHDA-Containing Liposomes 

Liposomes were prepared by dissolving 1.6 mg DMPC (1,2-dimyr-istoyl-sn-glycero-3-phosphocholine) in 200 μL chloroform, followed by evaporation under an air stream. Then, 1.5 mL distilled water was added (or 0.97 mM aqueous CHDA solution, for preparation of liposomes with CHDA) and sonicated for 3 min. The sample was then frozen in liquid nitrogen, thawed four times to contract the volume of the liposomes, and dialyzed against 1 L H_2_O for 24 h at room temperature, changing the water 3 times. Then, the samples were extruded through a 100 nm pore membrane. The CHDA content in the liposomes was determined by UV–VIS spectroscopy. The liposome solutions were dried and redissolved in ethanol. The absorbance was measured at 256 nm to determine the concentration and calculate the CHDA loading density.

### 3.3. In Vitro Studies

#### 3.3.1. Inhibition of the Enzyme Acetylcholinesterase (AChE)—IC50 Determination

The activity of the AChE enzyme isolated from *Electrophorus electricus* (Sigma-Aldrich, St. Louis, MI, USA) and its inhibition after incubation with different concentrations of CHDA, liposomal CHDA, and rivastigmine (Sigma-Aldrich) were measured using a modified Ellman spectrophotometric method [42]. IC50 determination was carried out in two steps as follows: (1) after a 10 min incubation of 20 µL of AChE enzyme (185 U/L) with 10 µL of an aqueous solution of CHDA, liposomal CHDA, or rivastigmine, and (2) after the reaction of AChE for 10 min with 135 µL of a solution containing acetylthiocholine iodide (ACTI; 0.45 mM), 5,5’-dithio-bis-2-nitrobenzene acid (DTNB; 0.30 mM), and phosphate buffer at 37 °C, pH = 8. AChE inhibition under rivastigmine was measured in the concentration range of 0–0.15 mM, while CHDA and liposomal CHDA were measured in the range of 0–1.5 µM. Absorbance was measured at 412 nm for 10 min at 1 min intervals using a 96-well microplate reader (Epoch, BioTek Instruments, Winooski, VT, USA). Absorbance was measured for each concentration of inhibitor (rivastigmine, CHDA, liposomal CHDA). The Δ absorbance values were presented relative to the logarithm of the inhibitor concentration, determining the IC50. The latter represents the inhibitor concentration that causes 50% inhibition of the enzyme.

#### 3.3.2. Formation of Amyloid β 1-42 (Aβ42) Fibrils

Aβ42 fibrils are the main component of amyloid plaques in the brain of people with AD, while Aβ40 is only detected in a subset of plaques, so Aβ42 was chosen for further study. Amyloid fibrils were formed from human amyloid β 1-42 peptide (rPeptide, Bogart, GA, USA) according to the protocol of Stine et al. [43]. The lyophilized powder was pretreated with hexafluoroisopropanol (HFIP), and the peptide film formed in the tube was dissolved in DMSO to a concentration of 5 mM. For fibrillization, the Aβ solution was diluted to a concentration of 100 μM in 10 mM HCl (pH 2.2) and incubated for 3 days at 37 °C. The formation of fibrillar Aβ42 aggregates was confirmed by atomic force microscopy (AFM).

#### 3.3.3. Dissociative Activity of Tested Formulations—Thioflavin T (ThT) Fluorescence Assay

All constituents, including CHDA, were tested beforehand for possible interaction with ThT to avoid false positive/negative results (Figure S1, Supplementary Materials). In order to determine the dissociation activity of the tested formulations, a solution of Aβ42 fibrils was diluted in 10 mM phosphate buffer (pH 7.4) to a working concentration of 5 µM and incubated for 24 h with the tested formulations in the concentration range of 100 pM to 100 μM. After a 24 h incubation of amyloid fibrils with the tested formulations, the solution of ThT in phosphate buffer (10 mM, pH 7.4) was added to the samples at a final concentration of 50 μM to maintain a fibril–ThT ratio of 1:10 and then incubated for 60 min in the dark at 37 °C. Fluorescence intensity in the 465–600 nm range was detected after excitation at 440 nm using a Synergy Mx spectrofluorometer (BioTek Company, USA). All measurements were performed in triplicate, and the final value was the average of these three independent samples normalized to the fluorescence intensity of Aβ42 fibrils alone (taken as 100%) with standard deviation. The averaged data values were fitted using Sigmoidal Equation 4 in SigmaPlot 12 software (Systat Software Inc., Chicago, IL, USA). Dissociation activity is represented by DC50 values (concentration of compound with 50% dissociation efficiency). 

#### 3.3.4. Effect of CHDA on the Degradation of Aβ42 Fibrils—Atomic Force Microscopy (AFM)

The dissociative effects of the compounds on Aβ42 fibrils were examined using a Innova Atomic Force Microscope (Veeco, Plainview, NY, USA) in tapping mode with an SNL-10 tip (Bruker, Billerica, MA, USA). The samples of Aβ42 fibrils (5 µM) incubated for 24 h with 40 µM of the test formulations were diluted 1:2 with ultrapure H_2_O. A drop of the sample was transferred to a clean mica surface and incubated for 5 min. After adsorption, the surface was washed with ultrapure water and dried under N_2_ gas. The scanning frequency was set at 0.5 Hz with a resolution of 1024 × 1024 pixels. Images were processed and evaluated using Gwyddion 2.55 software.

#### 3.3.5. Effect of CHDA on Neural Cell Viability—Cell Culture and Counting

Human SH-SY5Y neuroblastoma cells (ECACC 94030304; Sigma Aldrich, St. Louis, MO, USA) were cultured in DMEM medium (Sigma Aldrich Company) with 2 mM L-glutamine, 100 IU/mL penicillin, and 100 mg/mL streptomycin and supplemented with 10% fetal bovine serum (FBS). Cells were maintained under standard conditions, at 37 °C in an atmosphere of 95% humidity and 5% CO_2_ content. The effects of the CHDA, liposomal CHDA, rivastigmine at a concentration of 10 µM, and liposomes test formulations on the SH-SY5Y neuroblastoma cell line were examined using the WST-1 assay (Roche Diagnostics, Basel, Switzerland, 05 015 944 001). The WST-1 assay protocol is based on the cleavage of the tetrazolium salt of WST-1 to formazan by mitochondrial cellular dehydrogenases. The higher the number of living cells, the higher the activity of mitochondrial dehydrogenases and thus, the greater the amount of formazan dye formed. Cells were spread in 96-well plates at 7 × 10^4^ cells per well in 10% DMEM medium. After overnight incubation at 37 °C and 5% CO_2_, the test compounds dissolved in H_2_O at a concentration of 10 µM were added to the wells and incubated for an additional 24 and 48 h. WST-1 reagent was then added to the cell culture medium and incubated for 15 min at 37 °C. Absorbance was measured at A_450nm_–A_600nm_ using a microplate reader. Data were expressed as a percentage of cell viability. Cell viability was calculated using the formula:

% cell viability = (absorbance of formulation − 0.045)/(absorbance without formulation − 0.045) × 100.

### 3.4. In Vivo Studies

#### 3.4.1. Animals and Study Groups—Experimental Procedure

Adult male Wistar rats weighing 190–200 g (line Cmd: (WI) WU) were used for toxicity studies of test formulations. Experimental procedures on animals were approved by the 2nd Warsaw Ethical Committee (permits no.: WAW2/134/2019 and WAW2/052/2022). The rats were housed in a temperature-controlled (22 ± 2 °C) and humidity-controlled (55 ± 5%) room with a 12 h light/dark cycle and were given free access to food and water. For seven consecutive days, the rats were injected intraperitoneally (ip) with (i) a physiological NaCl solution (0.9%) as the control (n = 6), (ii) 1.5 mg/kg CHDA dissolved in NaCl (n = 6), (iii) 1.5 mg/kg CHDA administered in liposomal form (n = 6), and (iv) the reference compound rivastigmine at a dose of 1.5 mg/kg. Then, 24 h after the last injection, the rats were deeply anesthetized with pentobarbital sodium (Biowet, Pulawy) at a dose of 80 mg/kg administered intraperitoneally. Blood samples for gas measurements were taken from the carotid artery, and blood samples for morphological and biochemical analyses were obtained from cardiac puncture. After euthanasia with a supplemental dose of pentobarbital (80 mg/kg), urine samples were collected by bladder puncture.

#### 3.4.2. Toxic Effects on Organs as a Result of Repeated Exposure—Blood and Urine Analysis

Morphological and biochemical parameters of blood collected after seven days of animal exposure to CHDA, liposomal CHDA, rivastigmine, and NaCl were analyzed using an XT-2000i hematology analyzer (Sysmex, Kobe, Japan) and a Cobas 501 photometric device (Roche) at ALAB Veterinary Laboratory. Arterial blood gas analysis was carried out with a VetStat electrolyte and blood gas analyzer (IDEXX Laboratories, Westbrook, ME, USA). Rat urine analysis was completed using Siemens Multistix 10SG Professional Urine Reagent Test Strips (Siemens Healthcare, Warsaw, Poland).

#### 3.4.3. Determination of Acetylcholinesterase (AChE) Activity in Hippocampal Homogenates after Injection of CHDA, Liposomal CHDA, and Rivastigmine

The rats were sacrificed with sodium pentobarbital (250 mg/kg ip) 50 min after the intraperitoneal injection of NaCl, CHDA, liposomal CHDA, and rivastigmine or the intravenous administration of CHDA and liposomal CHDA dissolved in physiological NaCl solution (at a dose of 1.5 mg/kg). The rats’ brains were then isolated, and the hippocampi were harvested and frozen at −80 °C. The frozen tissue was homogenized in lysis buffer (50 mM TRIS-HCl solution with 150 mM NaCl, 1% Triton X-100, pH = 8) using an ultrasonic homogenizer. A total of 20 mg of tissue per 1 mL of buffer was used. The prepared homogenates were centrifuged at 14,000 rpm for 10 min at 4 °C. The supernatant was then used to determine AChE activity. AChE activity in the samples was quantitatively measured by the Ellman method [42], with modification, using a 96-well microplate reader (Epoch. BioTek). A mixture of 0.4 mL of supernatant with 2.6 mL of DTNB solution (10 mM) was prepared. A total of 155 µL of the mixture was transferred to a 96-well plate in triplicate for each sample. The plate was then incubated for 10 min at 37 °C. After incubation, 10 µL of ACTI (7.5 mM in distilled water) was added to each well, and the absorbance was measured at 412 nm for 10 min at 1 min intervals. AChE activity was determined using a calibration curve made at each measurement with acetylcholinesterase from *Electrophorus electricus* (Sigma-Aldrich) and expressed in mU min^−1^ mg^−1^.

### 3.5. Statistical Analysis 

Statistical analysis was performed using Statistica, version 12 (StatSoft Inc., Tulsa, OK, USA). The normality of the distribution was tested with the Shapiro–Wilk test. Data were reported as means ± SDs. Differences in mean values between investigated groups were analyzed by the U Mann–Whitney test for independent variables. *p* < 0.05 was considered to indicate significant differences. 

## 4. Conclusions

In the present work, we showed that the new tetrahydroacridine derivative CHDA is, compared with rivastigmine, a very potent AChE inhibitor with low toxicity to nerve cells and in studies on living organisms. In addition, it exhibits dissociative activity against amyloid β fibrils, a highly desirable property in the context of applications for the potential therapy of Alzheimer’s disease. Unfortunately, the tested compound appears to cross the blood–brain barrier to a limited extent, which is not facilitated by its incorporation within liposomes. In view of the aforementioned favorable features of the CHDA compound, further studies are needed to improve the compound’s ability to penetrate the BBB.

## Figures and Tables

**Table 1:** Analysis of blood from rats administered intraperitoneally with physiological NaCl solution (0.9%), CHDA, liposomal CHDA, and rivastigmine (reference compound), for seven consecutive days. Hematological data: red blood cells (RBCs), white blood cells (WBCs), hemoglobin (HGB), hematocrit (HTC), mean corpuscular volume (MCV), mean hemoglobin (MCH), mean hemoglobin concentration (MCHC), and platelets (PLTs). Blood biochemical analysis: alanine aminotransferase (ALT), aspartate transaminase (AST), blood urea nitrogen (BUN), and creatinine (CREA). Arterial blood gas analysis: pH, arterial blood carbon dioxide partial pressure (pCO_2_), arterial blood oxygen partial pressure (pO_2_), bicarbonate level (HCO^3−^), hemoglobin oxygen saturation (SaO_2_), base excess (BE), total hemoglobin (tHb), sodium level (Na^+^), potassium level (K^+^), and chloride level (Cl^−^).

	NaCl (n = 6)	Rivastigmine (n = 6)	Liposomal CHDA (n = 6)	CHDA (n = 6)
**Blood morphology**				
**RBC [T/L]**	8.6 ± 0.4	7.7 ± 0.4	8.00 ± 0.5	8.6 ± 0.6
**HGB [g/dL]**	16.8 ± 1	15.37 ± 1 *	15.8 ± 1	17.1 ± 0.5
**HTC [%]**	49.5 ± 5.7	45.2 ± 2.7	46.8 ± 2.2	49.2 ± 1.2
**MCV [fL]**	55.9 ± 1.8	58.4 ± 1.9	58.7 ± 2.7	56.9 ± 2.3
**MCH [pg]**	19.6 ± 0.6	19.8 ± 0.5	19.8 ± 1.2	19.8 ± 1
**MCHC [g/dL]**	35.1 ± 0.7	33.97 ± 0.5	33.8 ± 0.6	34.7 ± 0.5
**PLT [G/L]**	1065 ± 67	956 ± 163	1099 ± 94	1038 ± 171
**RDW-CV [%]**	16.4 ± 1.1	12.6 ± 0.7 *	12.8 ± 1.8 **	17.3 ± 1.6
**WBC [G/L]**	9.6 ± 1.4	6.3 ± 1.5	14.4 ± 14	9.9 ± 2.8
**Biochemical analysis**				
**ALT [U/L]**	54.7 ± 9	38.7 ± 20	47.3 ± 8	59.2 ± 7
**AST [U/L]**	132 ± 36	112.25 ± 28	167.83 ± 53	142.25 ± 25
**BUN [mg/dL]**	44 ± 9	44.00 ± 5	54.16 ± 7	43.5 ± 3
**CREA [mg/dL]**	0.33 ± 0.08	0.36 ± 0.14	0.31 ± 0.08	0.35 ± 0.06
**Arterial blood gas analysis**				
**pH**	7.38 ± 0.08	7.42 ± 0.07	7.36 ± 0.03	7.36 ± 0.07
**pCO_2_ [mmHg]**	49 ± 12	42.5 ± 9	48.7 ± 5	46 ± 9
**HCO_3_^−^ [mmol/L]**	25.8 ± 2.5	25.3 ± 1.7	25.5 ± 1.3	23.7 ± 1.73
**AnGap [mmol/L]**	12.2 ± 1.5	13.5 ± 0.6	13.7 ± 0.9	12.6 ± 0.84
**tCO_2_ [mmol/L]**	27.3 ± 3	26.6 ± 2	27 ± 1.4	25.1 ± 2
**BE [mmol/L]**	0.01 ± 1.5	1.2 ± 1.2	−0.3 ± 0.9	−2.3 ± 1.7
**pO_2_ [mmHg]**	88.5 ± 24	80.3 ± 12	82.5 ± 9	84 ± 21
**tHb [g/dL]**	17.2 ± 1.6	16 ± 0.7 *	16 ±0.7	17.6 ± 0.4
**SaO_2_ (%)**	92.5 ± 5	93 ± 3	91 ± 3	90.6 ± 5
**Na^+^ [mmol/L]**	139.6 ± 2	142 ± 2	143 ± 1	138 ± 1
**K^+^ [mmol/L]**	5.08 ± 0.9	5.8 ± 0.8	5.2 ± 0.6	6.0 ± 0.5
**Cl^−^ [mmol/L]**	106 ± 2	109 ± 2	109 ± 1	107 ± 1

* *p* < 0.05, ** *p* < 0.01 compared with the CHDA group.

**Table 2:** Urine analysis of rats administered intraperitoneally with physiological NaCl solution (0.9%), CHDA, and CHDA in liposomes and rivastigmine (reference compound), for seven consecutive days. Measured parameters: glucose (GLU), bilirubin (BIL), ketone bodies (KET), specific gravity (SG), blood (BLO), pH, protein (PRO), urobilinogen (URO), nitrites (NITs), leukocytes (LEUs).

Parameter	NaCl (n = 6)	Rivastigmine (n = 6)	Liposomal CHDA (n = 6)	CHDA (n = 6)
**GLU [g/L]**	negative	negative	negative	negative
**BIL**	negative	negative	negative	negative
**KET [g/L]**	negative	negative	negative	negative
**SG [g/mL]**	1.017 ± 0.003	1.014 ± 0.002	1.016 ± 0.002	1.016 ± 0.002
**BLO [µL^−1^]**	negative	negative	negative	negative
**pH**	7.75 ± 0.27	8.2 ± 0.27	7.67 ± 0.47	8.05 ± 0.27
**PRO [mg/dL]**	30–100	30–100	30–100	30–100
**URO [mg/dL]**	0.2	0.2	0.2	0.2
**NIT**	negative	negative	negative	negative
**LEU [µL^−1^]**	negative	negative	negative	negative

Statistical analysis showed no significant differences among the study groups.

## Data Availability

The data presented in this study are available in this article.

## Supplementary Materials

The following supporting information can be downloaded at: https://www.mdpi.com/article/10.3390/ijms251810072/s1.
