# Supplementary material for: A Novel Tetrahydroacridine Derivative with Potent Acetylcholinesterase Inhibitory Properties and Dissociative Capability against Aβ42 Fibrils Confirmed by In Vitro Studies
Source: Int J Mol Sci. 2024 Sep 19;25(18):10072. doi: 10.3390/ijms251810072 (PMC11444201; doi:10.3390/ijms251810072)
Supplement: Supplementary file 1 [file ijms-25-10072-s001.zip › ijms-3197317-supplementary.pdf]

Supplementary Materials

CHDA, liposomes and other constituents of the solutions were tested beforehand for possible interaction with ThT to avoid false positive/negative results of the assay. As seen from Figure S1A, CHDA absorb light in visible part of the spectra with maximum at 401 nm. The A_λ = 440 nm of CHDA at the highest used concentration (100 µM) is only 0.047 a.u. (Figure S1A, red X). Figure S1B shows ThT fluorescence intensities of untreated Aβ42 fibrils (100%, black column) and concentration range of CHDA (blue columns). ThT fluorescence of untreated Aβ42 fibrils is significantly higher compared to CHDA + ThT reference values. Furthermore, we did not observe interference of CHDA with ThT fluorescence intensity values (Figure S1B, C), and the increase in fluorescence value (3903 → 4131 f. u. (red Xs in Fig. S1C, D)), even though within instrument deviation, results from the slight change of the spectra in the presence of the unbound ThT. Reference fluorescence values of CHDA + ThT were adequately subtracted from samples' fluorescence values.

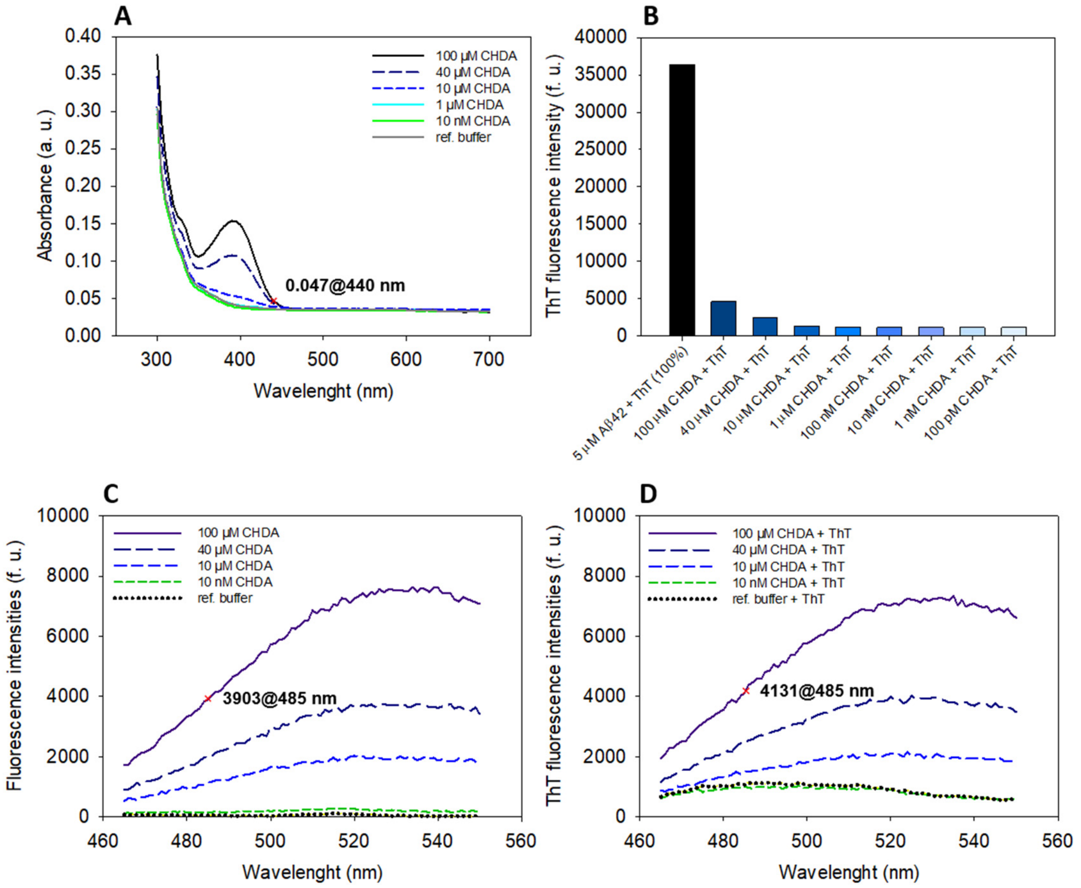

Figure S1: (A) Absorbance spectra of CHDA in relevant concentration range; highlighted absorbance within the graph correspond to λ_A = 440 nm. (B) Difference between ThT fluorescence intensities of untreated Aβ42 fibrils (100%) and concentration range of CHDA. Fluorescence intensities of CHDA (C) without and (D) in the presence of ThT. The excitation wavelength was λ_{em} = 440 nm, and highlighted fluorescence values within graphs correspond to λ_{ex} = 485 nm.
